# Death from Nitrous-Oxide Gas

**Published:** 1894-06

**Authors:** Frank J. Thornbury

**Affiliations:** Demonstrator of Bacteriology, University of Buffalo, N. Y.


ARTICLE IV.

### DEATH FROM NITROUS-OXIDE GAS.

BY FRANK J. THORNBURY, M. D.

Demonstrator of Bacteriology, University of Buffalo, N. Y.

Nitrous-oxide (nitrogen monoxide, "laughing gas," protoxide o-nitrogen) is a colorless, almost odorless, gas, made by the distillation of ammonium nitrate at a temperature of from 460° to 480° F.

When pure nitrous oxid gas is inhaled for from half a minute to three minutes, insensibility is produced, preceded in many instances by much excitement. Some persons under the influence of the gas sink quietly into unconsciousness, while others become hilarious, erotic, or pugnacious. During anæsthesia from this agent, patients present evidence of asphyxia, although the phenomena of mechanical asphyxia are different from those produced by nitrous-oxide gas. The cerebral pulsations are decidedly lessened and finally abolished, although the cerebral pressure is increased.

The causes of the effect of nitrous-oxid upon the system are not positively understood; the phenomena produced seem to be due to deprivation of the blood of oxygen. One observer (Prof. Paul Bert), believing that the production or non-production of anæsthesia by nitrous oxide is due to the tension of the gas in the blood, devised an apparatus by means of which the nitrous-oxid could be administered to the patient mixed with an equal quantity of oxygen, under a pressure of at least two atmospheres; the result is said to be satisfactory. The method, although expensive and cumbersome, is much used in Paris. Smaller animals live in an atmosphere of nitrous-oxid gas for only short intervals, the time varying from thirty seconds for the mouse, to sixty minutes for the frog. All finally die of asphyxia. Life, however, can be sustained in the gas by introducing a quantity of oxygen, approximating that ordinarily contained in air.

The circulatory changes in asphyxia, due to deficient oxygenation of the blood, are at first peripheral. The dark, non-ærated blood refuses to circulate through the capillaries, and a damming back and subsequent paralysis of the heart take place. During nitrous-oxid narcosis the amount of carbon dioxide exhaled from the lungs is much below the normal. This anæsthetic has been administered to hundreds of thousands of cases and but very few deaths are recorded. Only two or three fatal cases are mentioned in most of our standard text-books. The administration by means of an India-rubber bag containing at least eight gallons is the preferable method. The mouth-piece should contain two valves so that the expired gas is thrown out into the air and not back again into the bag.

Following this prelude to the use of nitrnus-oxid, I desire to make a brief report of an autopsy, made May 1st, for the coroners of Erie County, upon a woman who died after the administration of four gallons ot nitrous-oxide by a dentist for the extraction of four teeth. Soon after the induction of anæsthesia, the patient began to show signs of embarrassed breathing. Medical consultants were summoned. The pulse became rapid and attempts at breathing spasmodic. Artificial respiration was resorted to; the lower extremities were elevated. Nitro-glycerin, gr. 1-100, was administered, hypodermatically, and ammonia applied to the nostrins. The patient seemed to rally for a short time, but unconsciousness continued; the pulse became more rapid and feeble, and the heart's action finally ceased.

The report of the autopsy was as follows: The body is that of a middle-aged female, fairly developed and poorly nourished. Post-mortem rigidity is slight. Post-mortem staining is present over the pendant portions of the body. The ears are filled with cotton. The median incision shows the subcutaneous fatto be small in amount. No pleuritic adhesions exist in thethorax. The right lung weighs sixteen ounces; is universally congested and edematous; there is exudation of a large quantity of frothy serum. The left lung weighs thirteen and a half ounces, and is also congested and edematous. The lining membrane of the bronchi upon both sides are intensely hyperemic, and the tubes partially filled with frothy exudate. The heart weighs ten ounces and is of normal size and color. The ventricles are firmly contracted, and the walls of the left ventricle are slightly thickened. There is a dark clot in the chorda of the mitral valve. There are three minute atheromatous areas on the posterior wall of the aorta just above the segments of the valve. Over the anterior surface of the esophagus are two areas of hemorrhagic extravasation, irregular in outline and about the size of a half-dollar. The tracheal mucosa is heavily coated by a frothy, removable mucus. The larynx is congested and the lining membrane is also covered with frothy, slightly blood-tinged mucus. The congestion is especially marked in and just below the ventricles. The aryteno-epiglottidean folds are considerably corrugated, suggesting pre-existing edema. The position of the viscera is normal. The spleen is dark, moderately firm, normal in size and in consistency. The kidneys show dark-bluish discoloration; the surface is smooth, the capsule non-adherent. The right is slghtly smaller than the left,and contains a blood-clot beneath the mucosa of the pelvis and the true renal substance. This clot occupied an area one half a square decimeter in extent. The ureters are normal and the bladder empty. No urine was obtained for analysis. The liver is normal, its weight three pounds. The stomach is normal in size and contains about six ounces of partially-digested food. The mucous membrane is coated with thick semi-tenacious mucus; the membrane is slightly slate-colored in appearance. The intestines are normal. The uterus shows catarrhal endometritis, and there is a small quantity of mucus in its cavity. The left ovary shows a very recent corpus luterum and there is also a small cyst present. No adhesions exist. The right ovary and the tubes are normal. The brain was not examined owing to inability to obtain the consent of the coroner.

This case illustrates the invariable presence of danger in the administration of anæsthetics, even of the supposed harmless "laughing-gas."

On the grounds of the pathology of use of oxygen in the treatment seems indicated, the condition of asphxia, as shown, being due to the deprivation of the blood of oxygen.—*The Medical News.*

---

Article V.

